# Defense response enhancement in strawberry via elicitors

**DOI:** 10.1007/s13205-016-0431-9

**Published:** 2016-06-08

**Authors:** Gihan M. H. Hussein, Tahany M. A. abdel-Rahman, A. H. Alwan

**Affiliations:** 1 Gene Transfer Lab, Plant Genetic Transformation Department, Agricultural Genetic Engineering Institute (AGERI), Agricultural Research Center (ARC), Giza, Egypt; 2 Botany and Microbiolgy Department, Faculty of Science, Cairo University, Giza, Egypt; 3 Biology Department, College of Science, AL-Mustansiriya University, Baghdad, Iraq

**Keywords:** Strawberry cell-suspension culture, Elicitors, Jasmonic acid, Salicyilic acid fa*wrky* gene

## Abstract

In this study, cell-suspension culture of strawberry (*Fragaria* × *ananassa*), cultivars Camarosa, and Sweet Charlie has been established. Embryogenic callus was induced by incubating the in vitro juvenile leaf explants on medium, containing 2-mg/l picloram at dark. Suspension culture was initiated from 4-week-old embryogenic calli in the liquid MS medium with 1-mg/l 2,4-D and 2-mg/l picloram. Suspension culture was maintained by sub-culturing each 3 weeks into a fresh medium. At week 9 after third sub-cultures, torpedo and cotyledonary embryo stages were observed. Embryos were then developed into shoots on medium 1 mg/l of each BA and IBA. Obtained shoots were successfully rooted on 1-mg/ml GA3, 0.5-mg/ml BA, and 1-mg/ml IBA. To enhance the resistance availability in strawberry plants, elicitation was applied by adding the JA and SA elicitors to the suspension culture with two doses (0.5 and 1 mM) individually and in combination, in addition to the fungal homogenate of *Macrophomina phasiolena* at concentration of 10^6^ spor/ml. The fa*wrky-1-Camarosa* gene, which has defense-related function, was detected in the different elicited strawberry tissues and isolated via RT-PCR. The isolated gene was submitted to GenBank with accession number (KX096885).

## Introduction

The cultivated strawberry (*Fragaria* × *ananassa* Duch.), a member of the *Rosaceae*, is the most economically important soft fruit worldwide (Parikka 2004; Debnath et al. 2007). Strawberry production loss is caused by several factors resulted from a complex interaction between abiotic (temperature, soil type, and moisture), and biotic (pathogen infections) factors. *Rhizoctonia* spp., *Pythium* spp, *Fusarium* spp., and *Macrophomina phaseolina* are the fungal species that associate with black root rot diseases which leading to limit fruit production worldwide (Browne et al. 2002; Millner 2006). In Egypt, strawberry root rot diseases causing by *M. phaseolina* (Maas 1998). In addition, it was found that *M. phaseolina* is the common fungal species with high abundance and wide distribution in strawberry plant parts in the Egyptian condition (Hussein et al. 2012).

Plants have evolved numerous complex defense mechanisms to survive of the fungal and microbial pathogen attacks. The plant pathogen resistance outcomes normally visible as necrotic spots, termed the hypersensitive response (HR). Hypersensitivity is the most powerful mode of plants resistance against pathogen attack (Song et al. 2003). HR is associated with the accumulation of salicylic acid and several classes of pathogenesis-related (PR) proteins, many of which exhibiting antimicrobial activity which is enhancing the plant defensive capacity against a broad spectrum of pathogens. This resistance is mostly expressed locally and in distal, uninfected tissues, which is known as systemic acquired resistance (SAR) (Klessig and Malamy 1994).

Complex signaling networks that involve protein kinase cascades are: transcription factors, other regulatory proteins, and pathogenesis-related (PR) genes (Tena et al. 2001; Pedley and Martin 2003). Many transcription factor genes are induced by pathogen infection or hormones associated with defense signaling (Mysore et al. 2002). Transcription factors bind specific *cis* elements of the promoters of many defense-related genes, then, activate their expression and enhance the plant’s ability to overcome disease (Singh et al. 2002). The major transcription factor families that have roles in defense are WRKY, ERF, bZIP, and MYB (Riechmann and Ratcliffe 2000; Singh et al. 2002). WRKY proteins have been characterized in diverse plant species, i.e., *Arabidopsis*, parsley, and tobacco, strawberry, and rice (Eulgem et al. 2000; Encinas-Villarejo et al. 2009; Nakayama et al. 2013).

The early defense features involve the production of signaling molecules: include reactive oxygen intermediates (ROIs; oxidative burst), jasmonate, nitric oxide, and salicylic acid (Odjakova and Hadjiivanova 2001; Delledonne et al. 2002).

Fungal infection, exogenously application of elicitors, such as salicylic acid (SA) and methyl salicylate (MeSA), and wounding are used to up-regulate the defense-related genes in plants (Shulaev et al. 1997; Durrant and Dong 2004).

Plant tissue culture is now a well-established technology, which has made significant contributions to the propagation and improvement of agricultural crops in general, in addition to greater contribution in the application of molecular biology. Understanding of the biological processes that permit the manipulation of in vitro morphogenesis and investigation on various physiological, biochemical, and molecular aspects of plant hormones is greatly advance to recognize and provide information that may help address the issues of in vitro recalcitrance or in vitro plant growth and development (Akin-Idowu et al. 2009).

Adventitious shoots regeneration in strawberry via organogenesis has been previously reported from different explants, such as leaves (Debnath et al. 2007; Zakaria et al. 2014) and petioles (Debnath 2005).

Cells suspension cultures can exhibit much higher rates of cell division than act cells in callus culture. Thus, cell suspension offers advantages when rapid cell division or many cell generations are derived, or when a more uniform treatment application is required (Kanwar et al. 2008). Few studies of producing somatic embryos in strawberry via cell-suspension culture have been reported. Somatic embryogenesis research with strawberries is still need more efforts to develop the technology (Graham 2005). Several hormones with different concentrations and combinations have been used for producing strawberry somatic embryogenesis. Wang et al. (1984) reported that medium containing 2,4-D (4.4 mg/l), BA (0.5 mg/l) and casein hydrolysate (500 mg/l) was the most effective medium for inducing strawberry somatic embryos. Whole plants were obtained from somatic embryos when transferred onto GA3 or BA plus NAA. However, embryogenic cultures maintenance was unsuccessful. In addition, embryogenic calli of strawberry was induced on BA and IBA (Donnoli et al. 2001). However, Biswas et al. (2007) found that NAA at 4 mg/l was the most efficient for leaf callus induction, while using medium supplemented with 1.0 mg/l 2,4-D, 0.5 mg/l BA, and 50 % proline was the best for somatic embryogenesis. According to Kordestanni and Karami (2008), reported that they produced somatic embryogenesis when leaves were cultured on medium containing 2-mg/l picloram. Husaini et al. (2008) developed a reliable and highly efficient somatic embryogenesis system of strawberry leaf explants on medium supplemented with TDZ at concentration of 4 mg/l. In study of Zakaria et al. (2014), adventitious shoot via direct organogenesis has been regenerated in three strawberry cultivars, i.e., Festival, Sweet Charlie, and Florida using the in vitro juvenile leaves explants on MS medium supplemented with 2 mg/l TDZ.

The aim of the present study was to develop a strawberry cell-suspension culture and exogenously apply the artificial elicitors (SA and JA) and fungal homogenate of *Macrophomina phaseolina*, for enhancing defense responses and isolating the defense-related gene *wrky-1-Camarosa* in strawberry.

## Materials and methods

### Plant materials

Suspension culture was conducted on the in vitro plantlets of strawberry cultivars (Sweet Charlie and Camarosa) that were kindly obtained from Modern Company (PICO).

Tissue culture condition: all experiments of cell-suspension cultures and regeneration of strawberry were carried out on MS medium (Murasighe and Skoog 1962), pH was adjusted at 5.8 before autoclaving. All plant cultures were maintained in a controlled growth chamber at 25 ± 2 °C under 8/16-h (dark/light) fluorescent lights.

### Methods

The in vitro strawberry plantlets were micropropagated on solidified medium supplemented with 1-mg/l gibbrillic acid (GA3), 0.5-mg/l benzyl adenine (BA), and 1-mg/l indol acetic acid (IAA) as recommended by Boxus (1999) and incubated for 4 weeks. Sub-culture was repeated on the same fresh medium each 4 weeks.

Embryogenic callus induction: the 4-week-old in vitro juvenile leaves as explants were cultured on callus induction medium and incubated at dark or light condition for 4 weeks. Three different solidified media were tested, MSCI supplemented with NAA at concentration of 1 mg/l (Biswas et al. 2007), MSCII containing 2-mg/l picloram (Kordestanni and Karami 2008), or MSCIII with NAA and picloram at concentration of 1 and 2 mg/l, respectively. Each treatment has ten plates (10 plants/plate). The fresh weight, size, color, and nature of calli were recorded.

Initiation of suspension culture: friable portions of the 6-week-old callus were cultured into 500-ml Erlenmeyer flasks containing a volume of 150-ml medium. Five different liquid media (Table 1) were tested to select an efficient suspension culture. It is worth mentioning that medium Sta3 has the same composition of callus induction medium MSCII, but supplemented with 6 % sucrose. Cultures were incubated under light condition and shaking at 110 rpm on orbital shakers (Gerhardt Model 4155 RO 500, 50 mm) for 3 weeks. Each treatment has five flasks each has three calli with a total calli number of 15 explants/treatment. This experiment was repeated three times. During these period, many single cells, clusters of cells, small- and big-aggregates are released from the callus into the suspension. To separate and maintain cell-suspension culture, mother suspension cultures were diluted ratio 1:1 to fresh media. This was performed using sterile meshes (0.5 mm) to collect single cells and the cell-aggregates.

**Table 1:** Composition of different media used for strawberry suspension culture

Media	Stal	Sta2	Sta3	Sta4	Sta5
2,4-D (mg/ml)	1	1	0	1	0
BA (mg/ml)	0.5	–	0	0	0
Picloram (mg/ml)	–	–	2	2	2
Sucrose (mg/ml)	60	60	60	60	30

The cell-suspension cultures were maintained by sub-culturing the 3-week-old suspension to fresh liquid media and incubating under shaking condition. Small yellowish and compact green calli were chosen and plated on the selected embryo development medium for plant regeneration.

Embryo development and plant regeneration: after three sub-cultures of cell suspension during 9 weeks, suspension cultures were filtered through 0.5-mm mesh and cultured onto 15 different solid media (Table 2). Each treatment composed of 10 Petri dishes, each had 10 cell-aggregates with the total number of 100 cell-aggregates, experiment was repeated for three times. Cultures were, then, incubated at dark for 1 week then transferred to the light. Sub-culturing was performed each 3 weeks.

**Table 2:** Composition of different media used for strawberry embryo development

Media	BA (mg/ml)	NAA (mg/ml)	GA3 (mg/ml)	IBA (mg/ml)	ABA (mg/ml)
Msl	**–**	**–**	**–**	**–**	**–**
Ms2	0.5	0.2	**–**	**–**	**–**
Ms3	0.1	0.1	**–**	**–**	**–**
Ms4	1.0	**–**	**–**	1.0	**–**
Ms5	**–**	**–**	1	**–**	**–**
Ms6	0.5	1	**–**	**–**	**–**
Ms7	0.5	**–**	**–**	**–**	**–**
Ms8	0.1	**–**	0.1	**–**	**–**
Ms9	1	1	**–**	**–**	**–**
Ms10	1	**–**	**–**	**–**	**–**
Ms11	1	0.5	**–**	**–**	0.25
Msl2	1	**–**	**–**	2	**–**
Ms13	0.5	**–**	**–**	1	**–**
Msl4	2	**–**	**–**	1	**–**
Msl5	0.5	**–**	1	1	**–**

Rooting stage: three media were tested to reach high root formation, MSR1 medium supplemented with 1-mg/ml GA_3_, 0.5-mg/ml BA, and 1-mg/ml IBA; MSR2 with 40-µg/ml NAA; and finally MSR3 containing 1-mg/ml GA3 and 1-mg/ml IBA. All shoots were incubated at light conditions for 3 weeks. The shoots did not form roots were re-transferred to a fresh rooting medium.

Elicitation treatment: jasmonic acid (JA) and salicyilic acid (SA) were added to the 4-week-old suspension culture at two concentrations (0.5 and 1 mM), individually and in combination. At the same time, autoclaved fungal homogenates of the *Macrophomina phaseolina* at concentration of 10^6^spor/ml was isolated and applied exogenously to the suspension culture of strawberry cultivar Camarosa. Thereafter, exogenous JA, SA, and *M. phaseolina*-elicited-strawberry callus tissues were harvested after 24-, 48-, and 72-h post-elicitation for detecting the fa*wrky-1-Camarosa* gene in different elicited tissues with the different elicitors.

#### Isolation, sequence, and alignment of gene fa*wrky1*gene

RT-PCR: to detect the expression of fa*wrky1* gene in the elicited strawberry tissues with different elicitors, total RNA of strawberry calli (Camarosa) obtained from different elicitation treatments was extracted as recommended by GF-1 total RNA extraction kit (Vivantis). The full length of the fa*wrky1* gene was isolated using specific primers WR-F “5′ATGGATACCTACCCAGCATTC3” and WR-R “5′TCACAAAGAAGTGTAGATTTGCAT3”, (EU727547). RT-PCR and the amplification reactions were performed by following the instruction procedure of the two-steps-RT-PCR kit (Vivantis), cDNA was produced with 2 µg of total RNA of strawberry.

Sequence and alignment: the obtained fragments of the different treatments were then purified and cloned into PGEM–T Easy vector and transferred to the cell *E. coli* of strain DH5 α. Screening of the transformed colonies was performed with *Eco*R1 digestion to choose the right colony carrying the gene of interest. The isolated DNA from each sample was then sequenced and all sequences using sp6 universal primer. Alignment was performed using http://www.ncbi.nlm.nih.gov/.

## Results

Embryogenic callus induction: the 4-week-old in vitro juvenile leaf explants started to form callus 10 days post-culturing on the three tested media, within 3 weeks, the embryogenic calli were produced. Thereafter, the produced calli were transferred to fresh media for more 3 weeks. During theses, 6 weeks calli were enlarged and be ready for transferring into suspension culture. Highest callus formation was observed on medium MSCII either at light or dark, followed by MSCI. Meanwhile, the lowest response on callus formation in both cultivars was observed on medium MSCIII (Table 3). Color and size of the produced callus were varied on the different culture media. Medium MSCI showed callus with light-to-moderate yellow greenish in color, and small-to-medium in size and compact. MSCII medium showed callus with moderate reddish, yellowish, greenish to brownish in color, medium and large in size, and friable, semi-friable to compact. Medium MSCIII produced reddish–yellowish color callus, small in size, compact to highly compact. Cultures under dark condition resulted in high fresh weight calli in both cultivars and all media (Table 3). In addition, it was observed that cultivar Camarosa showed more callus formation response than the cultivar Sweet Charlie (Table 3). Therefore, medium MSCII at dark was chosen to be the condition for strawberry callus induction.

**Table 3:** Callus induction of both strawberry cultivars on three different media under light or dark condition

Media	Light/Dark	Camarosa		Sweet Charlie	
		CIF %	Fresh weight g/calli	CIF %	Fresh weight g/3 calli
MSC1	Light	74	0.39	55	0.37
MSCI	Dark	85.2	2.5	80	2.3
MSCII	Light	81	0.45	79	0.43
MSCII	Dark	96	3.0	92	2.8
MSCIII	Light	70	0.11	41	0.16
MSCIII	Dark	75.0	0.9	60	1.2

*CFI* callus induction frequency

Initiation of strawberry suspension culture: the 6-week-old friable calli were transferred to the five different liquid MS media (Sta1, Sta2, Sta3, Sta4 and Sta5) and incubated under shaking and light condition for 3 weeks. Sub-culture was performed each 3 weeks under the same condition. Starting at week 5, the callus was dissociated into small cell-aggregates, and single cells were observed. At the 9th week, cell-suspension cultures obtained dark yellow to brownish calli, the cell-aggregates reached up to 2.5 mm, and the embryo stages (torpedo and cotyledonary) were observed (data not shown). Somatic embryogenesis frequency recorded upon cell-aggregates growth (increasing of cell-aggregates weight), percentage of callus produced embryos, and number of the embryos/3 ml suspensions. The frequency varied among the treatments, medium Sta2 containing 1 mg/l 2,4-D, and Sta4 containing 1-mg/l 2,4-D and 2-mg/l picloram gave higher percentage in both cultivars. However, media Sta1, Sta3, and Sta5 revealed low cell-aggregates weight and number of somatic embryo/3 ml suspension. In general, Camarosa cultivar recorded higher frequencies than the Sweet Charlie (Table 4).

**Table 4:** Somatic embryos frequency on different cell-suspension media

Media	Sweet Charlie			Camarosa		
	Cell-aggregates growth	Callus produced embryos (%)	No. of embryo/3 ml	Cell-aggregates growth	Callus produced embryo (%**)**	No. of embryo/3 ml
Stal	1.1	0	0	1.4	0	0
Sta2	1.8	87	24	2.1	90	28
Stt3	1.9	50	12	2.3	64	19
Sta4	2.7	82	21	3.1	88	27
Sta5	0.7	0	0	1.7	0	5

Somatic embryos development and regenerate whole plants: the cell-aggregates of different media were transferred to different solid media to develop into shoots. After seven weeks with three times sub-culturing, the embryos were developed into shoots. Embryo development into shoots frequency as well as number of shoots/aggregate revealed highest percentage with medium MS4 for both cultivars (Table 5). Embryogenic callus aggregates that obtained from the liquid medium Sta4 revealed higher frequency than those from medium Sta2. The conversion rate of embryo into shoots was at cultivar Camarosa 8 and 30 % in medium Sta2 and Sta4, respectively; however, in cultivar Sweet Charlie, it was 5 and 9 % in medium Sta2 and Sta4, respectively. Therefore, medium Sta4 for initiating suspension and medium MS4 for developing embryos and shoot regeneration were selected for cell-suspension protocol.

**Table 5:** Somatic embryo development and shoot regeneration frequency for strawberry cultivars Camarosa and Sweet Charlie on different media

Media	Sta2				Sta4			
	% no of embryo developed into shoots		Means of shoots/aggregate		% no of embryo developed into shoots		Means of shoots/aggregate	
	Camarosa	Sweet Charlie	Camarosa	Sweet Charlie	Camarosa	Sweet Charlie	Camarosa	Sweet Charlie
MS1	0	0	0	0	0	0	0	0
MS2	3	1	1	1	2	1	1	1
MS3	0	0	0	0	0	0	0	0
MS4	8	5	3	2	30	9	3	2
MS5	0	0	0	0	0	0	0	0
MS6	0	0	0	0	0	0	0	0
MS7	0	0	0	0	0	0	0	0
MS8	0	0	0	0	2	1	1	2
MS9	1	0	1	0	0	0	0	0
MS10	0	0	0	0	0	0	0	0
MS11	0	0	0	0	4	2	1.5	1.5
MS12	8	3	2.5	2	8	6	2	2
MS13	1	0	1	0	2	1	1	1
MS14	3	0	2	0	2	0	2	2
MS15	0	0	0	1	0	1	0	0

Rooting stage: medium MSR1 containing 1-mg/ml GA3, 0.5-mg/ml BA, and 1-mg/ml IBA showed highest root formation percentage in both cultivars (94 and 92 % for Camarosa and Sweet Charlie, in respect).

**Fig. 1:**
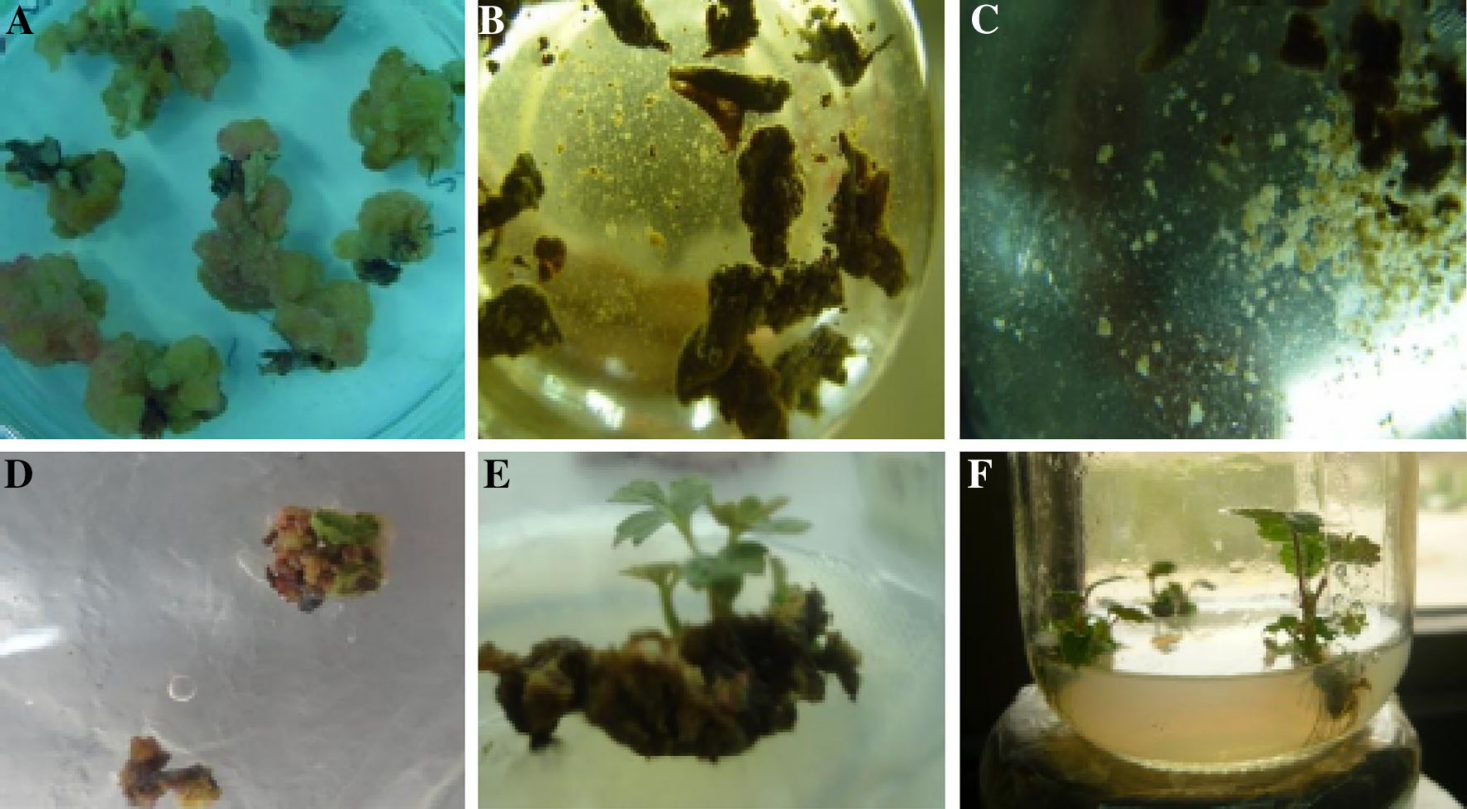
Somatic embryogenesis via cell-suspension culture in strawberry: **a** callus formation, **b** big cell-aggregates produced in cell-suspension culture, **c** small cell-aggregates and single cells in suspension culture, **d** developed embryo on medium MS4, **e** regenerated shoots on medium MS4, and **f** rooted shoots

Figure 1 illustrates somatic embryogenesis for strawberry cultivar Camarosa via the cell-suspension culture.

It can conclude the best condition for establishing the strawberry cell suspension as follows: embryogenic callus was induced by incubating juvenile leaf explants on medium MSCII containing 2-mg/l picloram, for 6 weeks at dark. Initiation, the cell suspension was carried out on the liquid medium Sta4 containing 1-mg/l 2,4-D and 2-mg/l picloram during three sub-cultures. Embryo was developed into shoots on the solid MS4 medium containing 1-mg/ml BA and 1-mg/ml IBA. Finally, medium MSR1 containing 1-mg/ml GA3, 0.5-mg/ml BA, and 1-mg/ml IBA was used for root formation.

### Isolation, sequence, and alignment of gene fa*wrky-1-Camarosa* gene

**Fig. 2:**
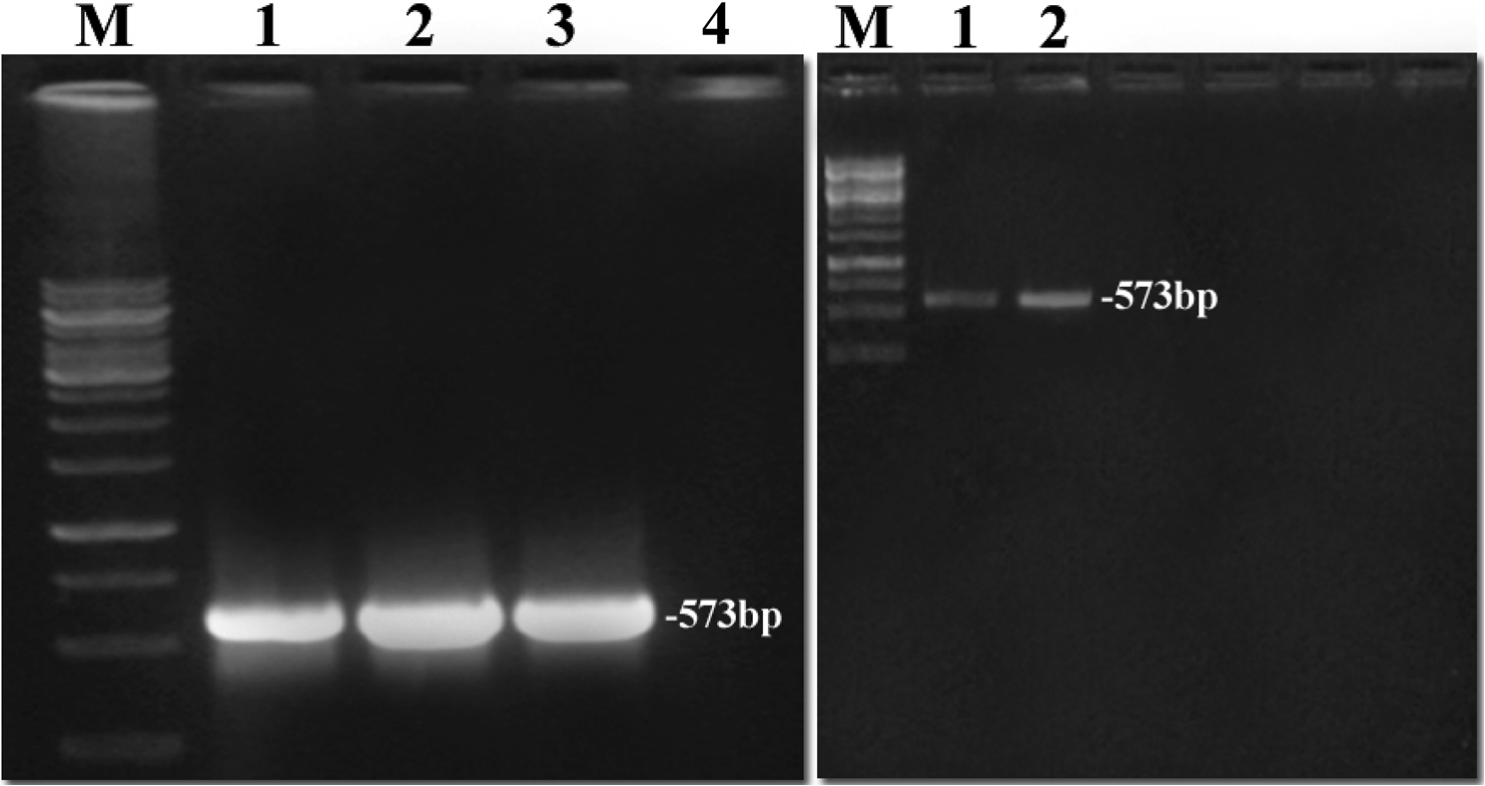
RT-PCR results of the elicited strawberry tissues with different elicitors and concentrations *right*: *M* ladder 1 Kb, elicited tissues with *1* 1.0-M SA/0.5-M JA, *2* 1.0 -M SA, *3* 1.0-M JA, and *4* negative control. *left*: *M* ladder 1 Kb, elicited tissues with *1* 1.0-M JA/0.5-M SA and *2* 0.5-M SA

RT-PCR was used for detecting the expression of the fa*wrky1* gene after elicitation. RT-PCR was carried out on the total RNA’s isolated from the nine elicited strawberry tissues with different treatments as well as the non-elicited tissues as negative control. Seven RNA samples were amplified fragment with a size of 573 bp represented seven different elicitation treatments (1.0-mM SA/0.5-mM JA; 0.5-mM SA/1.0-mM JA; 0.5-mM SA; 1.0-mM SA; 0.5-mM JA; 1.0-mM JA, and fungal homogenate 10^6^ spore/ml). The obtained fragment indicates the positive response of the elicitors and represents fa*wrky1* gene, whereas, no amplified fragment was detected with the RNA samples obtained from the non-elicited and elicited tissues with equal concentration of JA/SA (1.0/1.0 mM and/or 0.5/0.5 mM, Fig. 2).

**Fig. 3:**
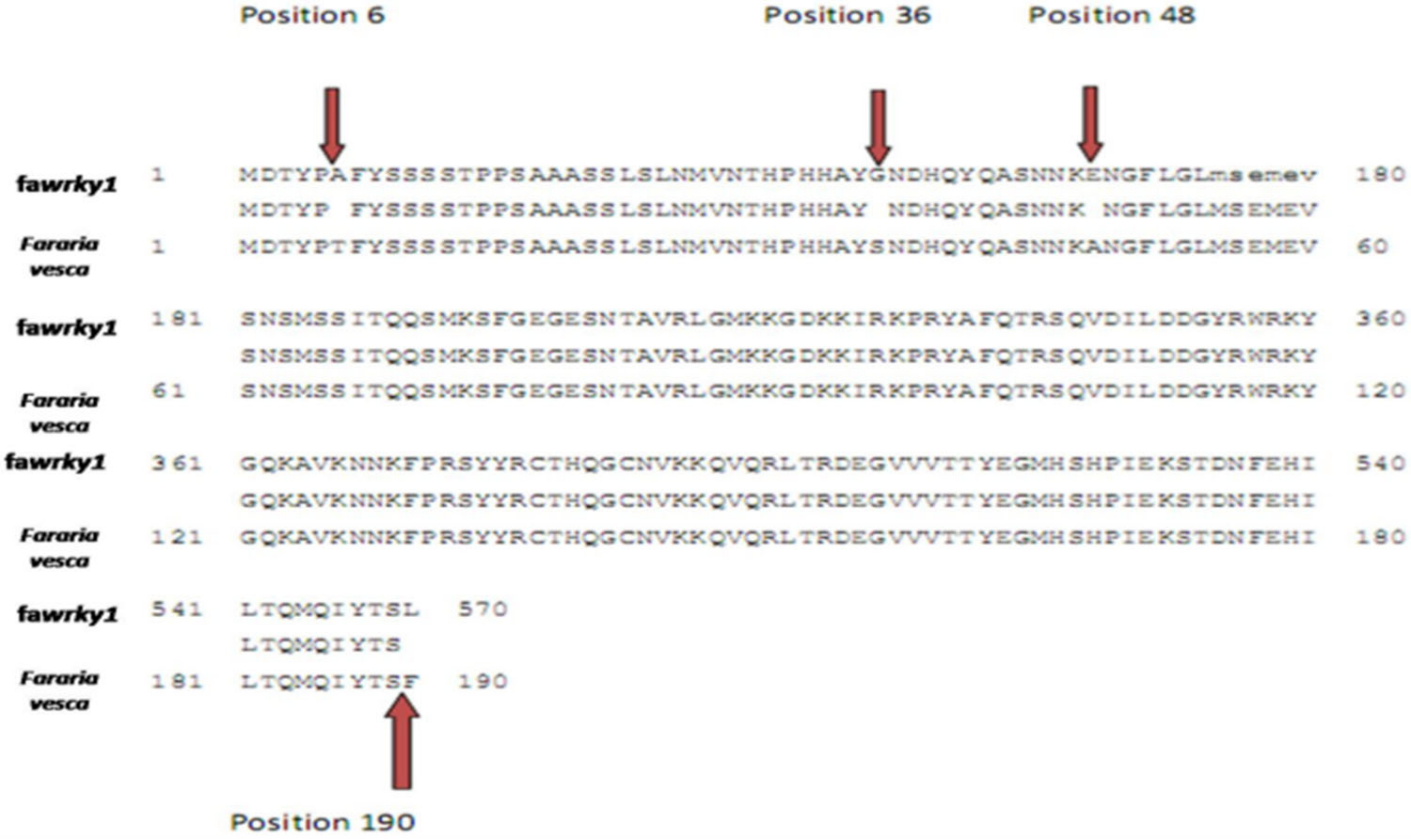
Blast x alignment shows 98 % homology with Fararia vesca subsp. Vescaprotein, 186 identical amino acids of a total 190. In faWRKY_1 (our sequence) Threonine (T) at position 6 was changed to Alanine (A), Serine (S) at position 36 was changed to Glycine (G), (A) was at position 48 changed to Glutamic acid (E) and Pheylalanine (F) to Leucine (L) at position 190

The obtained fragments of the different treatments were cloned in PGEM–T Easy vector and transferred to the cell *E. coli* strain DH5 α. Screening of transformed colonies was performed using *Eco*R1. The isolated DNA was then sequenced and aligned. Our sequence faWRKY_1-Camarosa was submitted to NCBI with accession number (KX096885). DNA alignment showed 99 % homology with* Fararia vescasubsp*.* Vesca* probable WRKY transcription factor 75. While it showed 98 % homology with* Fararia vesca subsp*. Vescaprotein, 186 identical amino acids of a total 190. In our sequence Threonine (T) at position 6 was changed to Alanine (A), Serine (S) at position 36 was changed to Glycine (G), (A) was at position 48 changed to Glutamic acid (E) and  Pheylalanine (F) to Leucine (L) at position 190 (Fig. 3).

## Discussion

Plant tissue culture and cell-suspension culture techniques are requested for determining how plants respond to their environment at the molecular level, including how plant cells defend themselves from pathogens (fungi, bacteria, viruses, etc.). Somatic embryogenesis is the process by which the somatic cells give rise to bipolar structure, and then develop to whole plants without gamete fusion (Iantcheva et al. 2005). Somatic embryogenesis used to initiate cell suspensions, which are used for many plant studies.

Present investigation demonstrates the successful strawberry plant regeneration via cell-suspension culture. The established protocol needs about 22 weeks to be completed, 6 weeks for callus induction, 9 weeks of suspension culture during, and finally, 6 weeks for embryo development.

High callus formation was induced on MSCII medium containing only picloram at concentration of 2 mg/l under dark condition. In general, two types of callus were formed, namely, friable- and semi-friable calli. Friable callus is preferable to use as inoculum for forming cell-suspension cultures that are because in friable callus, the cells are only loosely associated with each other and the callus becomes soft and breaks apart easily. In addition, dark condition is often applied for callus induction due to the lack of photosynthetic capability being no drawback (Quiroz-Figueroa et al. 2006).

In strawberry, a negative effect of light on somatic embryo induction has been previously reported in Clea (Donnoli et al. 2001). Similarly, Husaini and Abdin (2007) reported a dark treatment significantly increased the number of somatic embryos in the leaf explants in Chandler.

In the current study, the best frequency of cell-suspension culture was resulted on media containing either only 2,4-D or 2,4-D in combination with picloram (media, Sta2 and Sta4, respectively), whereas, 2,4-D combined with BA (medium Sta1) did not obtain any embryo. Moderate frequency of embryogenesis was recorded when using picloram alone (medium Sta3). Similar results were obtained by Kordestanni and Karami (2008) and Gerdakaneh and Zohor (2013) who reported that the 2-mg/l picloram yielded the highest percentage of embryonic calli in strawberry leaves of cultivars (Camarosa and Selva) and (Kurdistan, Paros, and Camarosa), respectively. However, another reports recommended other hormones for strawberry callus induction, such as 2,4-D in combination with BA (Wang et al. 1984); NAA (Biswas et al. 2007) and TDZ (Husaini et al. 2008).

Furthermore, sucrose as a carbon source concentration is one of the important factors in a plant cell culture (Dicosmo and Misawa 1995). This investigation showed that the sucrose concentration affected on the embryogenesis as sucrose at 3 % concentration (medium Sta5) did not obtain any embryos; however, the same medium composition with 6 % sucrose concentration (Sta3) revealed 50 % frequency. Higher sucrose concentration was previously recommended (Ricci et al. 2002; Karami et al. 2006; Kordestanni and Karami 2008) who reported that increasing sucrose concentrations may cause osmotic stress, but improved the somatic embryos development.

Embryo development and shoot regeneration, which is meaning developed the embryos at cotyledonary stage into whole plant, high frequency was successfully performed with calli obtained from Sta4 medium containing 2,4-D and picloram and on medium containing BA and IBA. Morphologically, normal plants were obtained from somatic embryos on 1-mg/l GA3 or 0.5-mg/l BA, and 0.1-mg/l NAA (Wang et al. 1984). However, Kordestanni and Karami (2008) found that the hormone free medium is the best for the embryo developments. Gerdakaneh and Zohor (2013) reported that 1-mg/l picloram was the best for yielding the highest number of cotyledonary-stage embryos.

The fungicides, bactericides, and insecticides, used in disease control, or their degradation products have harmfully affected the environment and human health. Therefore, searching for new, harmless means of disease control is strapping need. Genes-related defense were previously induced with the chemical elicitors. Elicitors are the compound chemical activates defense in the plants and inducing resistance to protect plants from pathogens. Chemical elicitors are commonly used are salicylic acid, methyl jasmonate, benzothiadiazole, and jasmonic acid. These chemical elicitors force the phenolic compound production and activate different defense-related enzymes in plants (Thakur and Sohal 2013).

It was previously reported that the common fungal species causing the root rot disease in strawberry, in Egypt, is *Macrophomina phaseolina* (Maas 1998 and Hussein et al. 2012). Therefore, autoclaved fungal homogenates of the *M. phaseolina* was isolated and applied exogenously to the strawberry suspension culture as a bio-elicitation and artificial elicitors for defense proteins enhancing.

Studies of the plant resistance to pathogens associated molecular in strawberry are limited. The first fa*wrky1* gene was isolated by Encinas-Villarejo et al. (2009) from strawberry cultivar Camarosa after acclimatizing 8-week-old in vitro plantlets in pots containing and grown for a minimum of 6 additional weeks prior to elicitor (ABA; SA) and wounding treatments or pathogen inoculation *Colletotri chumacutatum.* In this investigation, the fa*wrky1* gene was isolated from strawberry cultivar Camarosa after treating the in vitro suspension culture by different concentrations of the elicitors; JA, SA, and JA/SA, as it well known that the JA and SA are necessary for defense-related genes induction (Dempsey et al. 1999; Durrant and Dong 2004). In addition, autoclaved fungal homogenate of *M. phaseolina* was also applied. The fa*wrky1* gene was detected in all treatment except that with equal concentration of the JA and SA, indicating that gene fa*wrky1* is a factor-mediating defense response to *M. phasiolena* in strawberry. The current study declares that the gene fa*wrky1* has not been detected in the equal concentration JA/SA-elicited tissues. This may be due to the antagonistic and/or synergistic interaction between SA and JA pathways during cell death (Asai et al. 2000; Overmyer et al. 2000; Rao et al. 2000),which appeared when the two elicitors are applied in equal concentration. Likewise, SA has been shown to mediate the crosstalk between JA pathways (Spoel et al. 2003). Our isolated gene (fa*wrky1-Camarosa* KX096885) revealed 98 % homology with* Fararia vesca subsp.* Vescaprotein, 186 identical amino acids of a total 190. The fa*wrky1* sequence previously isolated by Encinas-Villarejo et al. (2009) who suggested that the fa*wrky1*encodes a IIc WRKY transcription factor and is up-regulated in strawberry following *C. acutatum* infection, treatments with elicitors, and wounding. They also reported that Fa WRKY1 sequence is homologue to At WRKY75 isolated from *Arabidopsis* and proved that Fa WRKY1 act as positive regulators of defense.

## Conclusion

The transcription factor Fa WRKY 1 which play role in the resistance to fungal pathogens was induced in strawberry by applying the artificial elicitors JA and SA, in addition to the autoclaved fungal homogenate of *M. phaseolina* in cell culture.
